# Local Tensile Stress in the Development of Posttraumatic Osteoarthritis

**DOI:** 10.1155/2018/4210353

**Published:** 2018-11-04

**Authors:** Dongyan Zhong, Meng Zhang, Jia Yu, Zong-Ping Luo

**Affiliations:** ^1^Orthopedic Institute, Medical College, Soochow University, Suzhou, Jiangsu 215006, China; ^2^Department of Orthopedics, The First Affiliated Hospital of Soochow University, Suzhou, Jiangsu, 215006, China; ^3^Suzhou Gusu District Women & Children Health Care Institution, Suzhou, Jiangsu, 215007, China; ^4^Huai'an First People's Hospital, The Affiliated Huai'an No. 1 People's Hospital of Nanjing Medical University, Huai'an, Jiangsu, 223300, China

## Abstract

The pathogenesis of posttraumatic osteoarthritis (PTOA) remains unrevealed. We speculate that cartilage crack caused by joint trauma will induce local abnormal tensile stress, leading to change in extracellular matrix (ECM) expression of chondrocytes, cartilage degeneration, and initiation of osteoarthritis. Finite element model was used to examine whether the local tensile stress could be produced around the crack. Cell experiments were conducted to test the effect of tensile strain on chondrocyte ECM expression. Animal tests in rabbits were carried out to examine the change around the cartilage crack. The results indicated that the local tensile stress was generated around the crack and varied with the crack angles. The maximum principal tensile stress was 0.59 MPa around the 45° crack, and no tensile stress was found at 90°. 10% tensile strain could significantly promote type I collagen mRNA expression and inhibit type II collagen and aggrecan (the proteoglycan core protein) mRNA expression. Type I collagen was detected around the 45° crack region in the cartilage with no change in type II collagen and proteoglycan. We conclude that the local tensile stress produced around the cartilage crack can cause the change in cartilage matrix expression which might lead to cartilage degeneration and initiation of osteoarthritis. This study provides biomechanical-based insight into the pathogenesis of PTOA and potentially new intervention in prevention and treatment of PTOA.

## 1. Introduction

Posttraumatic osteoarthritis (PTOA) is a common orthopedic disease that may occur after joint trauma. PTOA accounts for ~12% of all cases of osteoarthritis, which causes financial burden on the health care system [1, 2]. Until now, the pathogenesis of PTOA remains unrevealed [3].

Osteoarthritis is a chronic degeneration process involving the entire joint including the articular cartilage, subchondral bone, ligaments, capsule, and synovial membrane [4, 5]. The degeneration of cartilage and subchondral bone sclerosis is the main characteristic [6]. The main component of cartilage matrix is gradually changed from type II collagen and proteoglycan to type I collagen [7, 8]. Type II collagen fibers are arranged crosswise to form a network structure in which proteoglycans and other molecules are firmly bound together [9]. This sponge-like structure provides cartilage with the most important properties of withstanding the compression applied to joints during daily activities [10]. Type I collagen is the main component in bone, ligament, and tendon, which has enormous tensile strength needed in these structures [11]. This implies that a tensile stress environment may exist when osteoarthritis occurs causing the alteration of chondrocyte phenotype.

Based on these changes in cartilage structure and mechanical environment during cartilage degeneration of osteoarthritis, we propose an assumption of the pathogenesis of PTOA. Localized cartilage cracks may be produced after joint trauma, inducing abnormal tensile stress around the crack region; the alteration of local mechanical environment further causes changes in chondrocyte phenotype, downregulation of type II collagen and proteoglycan expression, and upregulation of type I collagen expression, leading to cartilage degeneration and initiation of osteoarthritis. The present study will verify this hypothesis both theoretically and experimentally. The results will provide a basic biomechanical support for future studies on the pathogenesis of posttraumatic osteoarthritis.

## 2. Materials and Methods

**Figure 1:**
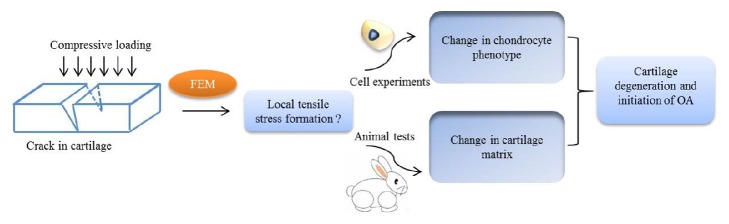
Flow diagram of the study design.

The study included three parts: finite element model (FEM), cell experiments, and animal tests. FEM was used to examine whether the local tensile stress could be produced around the crack. Cell experiments were conducted to test the effect of tensile stress on chondrocyte ECM expression. Animal tests were carried out to examine the cartilage change around the crack (Figure 1).

### 2.1. Finite Element Model

FEM simulated a two-dimensional cartilage layer. The cartilage thickness of 0.5 mm was from a typical New Zealand white rabbit sample used in the experiment and the length of the simulated crack was 0.3 mm. The elastic modulus and Poisson's ratio were 8 MPa and 0.42, respectively [12]. The intact cartilage was first simulated. The cracks were then analyzed at different angles from 15° to 90°. The surface loading was a uniform pressure of 0.15 MPa, simulating a normal loading to knee joint during daily walking [13].

### 2.2. Cell Experiments

#### 2.2.1. Isolation and Culture of Chondrocytes

Articular cartilage was isolated from knee joints of 4-month-old New Zealand white rabbits. Briefly, cartilage was aseptically removed, chipped and then minced. Diced tissue was digested in 0.2% type II collagenase (Sigma-Aldrich) for 3 hours at 37°C. The suspension was filtered through a 70 *μ*m nylon mesh. Chondrocytes were centrifuged and plated in culture medium [Dulbecco's modified Eagle medium (DMEM, Gibco) containing 10% fetal bovine serum (FBS, Gibco), 100 U/mL penicillin, and 100 *μ*g/mL streptomycin]. Passage 2 chondrocytes were used in subsequent experiments [14].

#### 2.2.2. Biomechanical Tests of Chondrocytes

**Figure 2:**
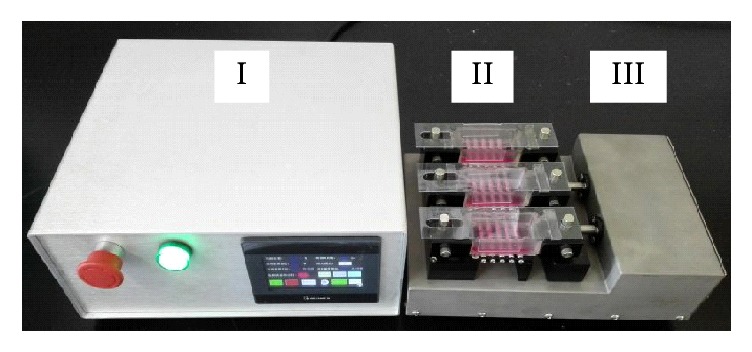
Custom-designed mechanical loading system. The system contains three parts: control unit (I), silicone chambers (II), and drive unit (III). The drive apparatus has one fixed end opposite an end which is driven by the control unit.

A custom-designed mechanical loading system was used to offer tensile strain (Figure 2). The system included three parts: a control unit, silicone chambers and a drive unit. The chambers were made of silicone elastomer, Sylgard® 184 (Dow Corning GmbH, Wiesbaden, Germany) with a surface measuring 3 × 6 cm. The chambers were sterilized and coated with fibronectin on the bottom surface before chondrocyte culture. One end of the chamber was attached to the drive device and driven by the control unit, and the other end was fixed. The arrangement enabled the entire silicon membrane area containing the cultured chondrocytes to be stretched uniformly. Chondrocytes at approximately 60% confluence were exposed to mild (5%) or excessive (10%) tensile strain at 0.5 Hz and 3 h/d for 3 days. Chondrocytes without mechanical loading were seeded onto the same dishes for use as a control.

#### 2.2.3. Total RNA Extraction and RT-qPCR

Total RNA was extracted from chondrocytes by using TRIzol reagent (Invitrogen, USA). 1*μ*g of total RNA was reverse-transcribed using a RevertAid First Strand cDNA Synthesis Kit (Thermo Fisher Scientific). To quantify mRNA expression, RT-qPCR was performed using the iTaq™ Universal SYBR® Green Supermix kit (Bio-Rad, Hercules, CA, USA) on a CFX96™ Real-Time PCR System (Bio-Rad). Relative mRNA expression levels of* COL1A1* (type I collagen),* COL2A1* (type II collagen),* Acan *(aggrecan), and* SOX9* were evaluated against* GAPDH* (glyceraldehyde-3-phosphate dehydrogenase) using the formula 2^-ΔΔCT^. The* C*T is determined from a log-linear plot of the PCR signal versus the cycle number and is an exponential term. Using the 2^-ΔΔCT^ method, the gene expression levels are presented as the fold change normalized to an endogenous reference gene* GAPDH* and relative to the untreated control [15]. The primer sequences were listed in Table 1.

### 2.3. Animal Tests

#### 2.3.1. Animal Model

The cartilage crack model was created using 15 four-month old New Zealand white rabbits weighed 2.5-3.0 kg. The study was approved by the Institutional Animal Care and Use Committee. The animals, housed in the animal care facility with free access to water and foods, were divided into 3 groups randomly. Anesthesia was achieved through injecting chloral hydrate (0.1 g/ml). The skin of their hind legs was shaved and disinfected with entoiodine for 3 minutes. The patella was then dislocated laterally to expose the fossa intercondyloidea of the femur. The crack was carefully made in the middle of the femoral trochlear groove using a scalpel, and the crack was angled in two directions: one perpendicular to the articular surface (90°) and the other oblique (45°). Each animal only received one crack angle. The joint was irrigated with 0.9% sodium solution after operation. The patella was repositioned and the capsule and skin were sutured. After operation, all the animals received antibiotics (penicillin) to prevent infection [16].

#### 2.3.2. Histology

The articular cartilage was evaluated at 2, 6, and 20 weeks. At each time point, the animals (n=5) were sacrificed via overdose of chloral hydrate. The knee joints were removed and fixed in 10% neutral buffered formalin for 2 days. The samples were then dehydrated and embedded in paraffin after decalcification in buffered 10% EDTA of pH7.4 for 4 weeks. Afterwards, horizontal plane sections of femoral trochlear groove (5 *μ*m) were cut into slides using a microtome (RM2165, Leica, Nussloch, Germany). For the evaluation of proteoglycans, slides were stained with safranin-O (Sigma-Aldrich Co, St. Louis, USA).

#### 2.3.3. Immunohistochemistry

Types I and II collagen in cartilage were evaluated immunohistochemically. Slides were deparaffinized in xylene and incubated with testicular hyaluronidase (2 mg/ml) (Sigma-Aldrich Co, St. Louis, USA) for 60 minutes at 37°C. The slides were treated with 1% H_2_O_2_ to inactivate endogenous peroxidase and 1.5% horse serum to eliminate nonspecific protein absorption. The sections were incubated with antibodies of type I and II collagen respectively (Abcam, San Francisco, USA). The samples were then exposed to the secondary antibodies (PK6200, Vector Laboratories, Burlingame, USA) and avidin. Color was developed with 0.3% diaminobenzidine tetrahydrochloride (Sk4100, Vector Laboratories, Burlingame, USA).

### 2.4. Statistical Analysis

All data were expressed as the mean ± standard deviation (SD). The differences between groups were analyzed by one-way analysis of variance (ANOVA) and Student's unpaired* t*-test, using SPSS 16.0 software (SPSS Inc., Chicago, IL, USA). Significance was indicated by a* P*-value of < 0.05.

## 3. Results

**Figure 3:**
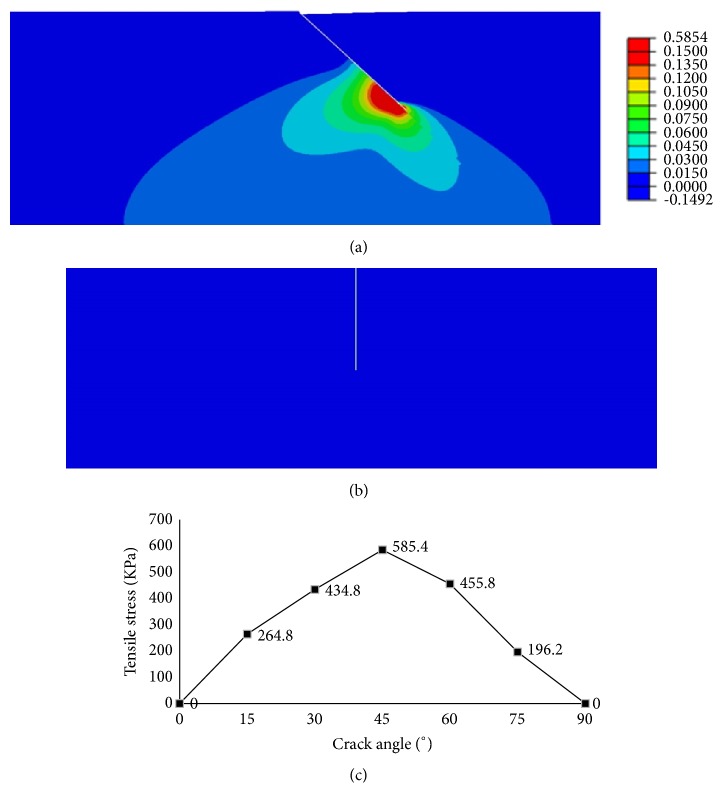
Finite element analysis. (a) The distribution of tensile stress around the 45° crack and the peak was 0.59 MPa. (b) The distribution of tensile stress around the 90° crack and no tensile stress existed. (c) The maximal tensile stress variation with the crack angle.

The finite element calculation indicated that cartilage crack could induce local tensile stress and the stress distribution around the crack changed significantly along with crack angles (Figure 3). In the intact cartilage, the maximal principal stress was uniformly compressive at 0.15 MPa. When the crack occurred, the tensile stress formed around the crack. A 45° crack induced the maximum tensile stress (0.59 MPa, or 3.9 times of the principal compressive pressure), while no tensile stress was found with a 90° cracking.

**Figure 4:**
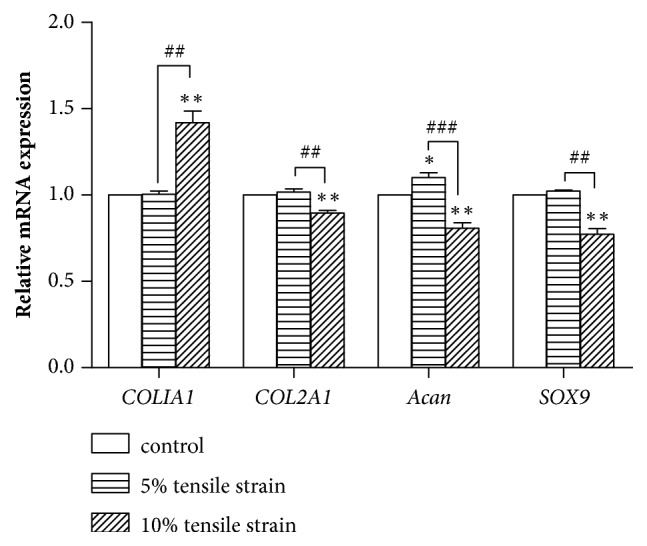
The mRNA expression levels of* COL1A1*,* COL2A1*,* Acan*, and* SOX9* (*n* = 3). *∗P* < 0.05, *∗∗P* < 0.01, and *∗∗∗P* < 0.001 compared with the control group. ^#^*P *< 0.05, ^##^*P *< 0.01, and ^###^*P *< 0.001 in the indicated groups.

Chondrocyte test* in vitro* showed that 10% tensile strain increased the expression of* COL1A1* by 41.9% and 41.2%, respectively, compared with the control group (*P* = 0.009) and 5% tensile strain (*P* = 0.006). Meanwhile, 10% tensile strain downregulated the expression of* COL2A1* by 11.7% and 12.0%, separately in contrast with the static group (*P *= 0.008) and 5% tensile strain group (*P *= 0.001), inhibiting the mRNA level of* Acan* by 20.3% (*P *= 0.009) relative to the untreated cells. In contrast to the control group, 10% tensile strain reduced* SOX9* mRNA expression by 22.8% (*P *= 0.007). The mild tensile strain (5%) had little influence on the expression of these genes (Figure 4).

**Figure 5:**
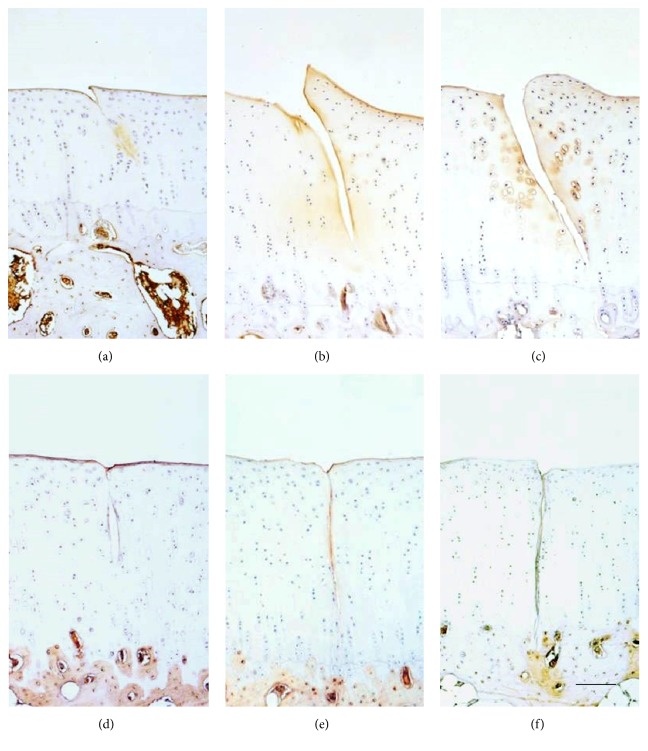
Immunohistochemical staining of type I collagen showed increase from 2 weeks to 20 weeks around the 45° crack but no presentation around the 90° crack. (a) 45° crack at 2 weeks. (b) 45° crack at 6 weeks. (c) 45° crack at 20 weeks. (d) 90° crack at 2 weeks. (e) 90° crack at 6 weeks. (f) 90° crack at 20 weeks. Scale bar: 200 *μ*m.

**Figure 6:**
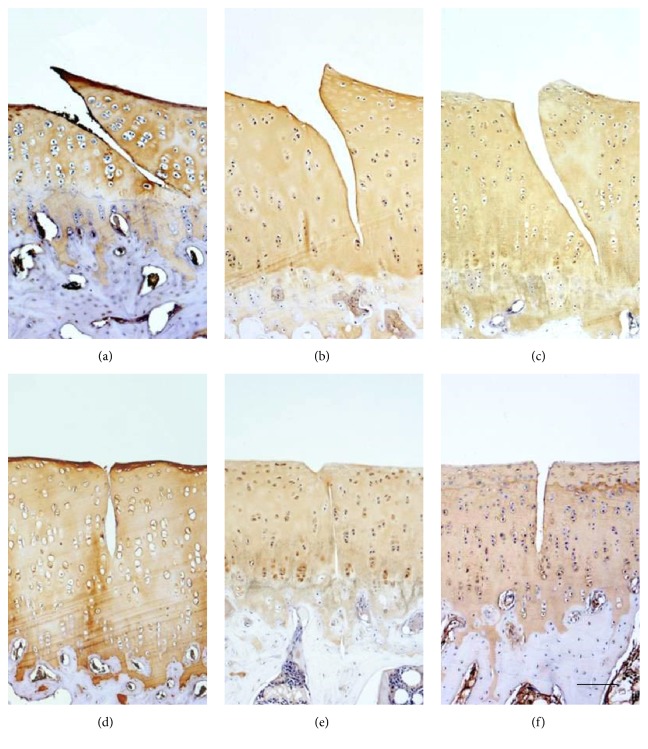
Immunohistochemical staining of type II collagen showed no significant changes from 2 weeks to 20 weeks. (a) 45° crack at 2 weeks. (b) 45° crack at 6 weeks. (c) 45° crack at 20 weeks. (d) 90° crack at 2 weeks. (e) 90° crack at 6 weeks. (f) 90° crack at 20 weeks.

**Figure 7:**
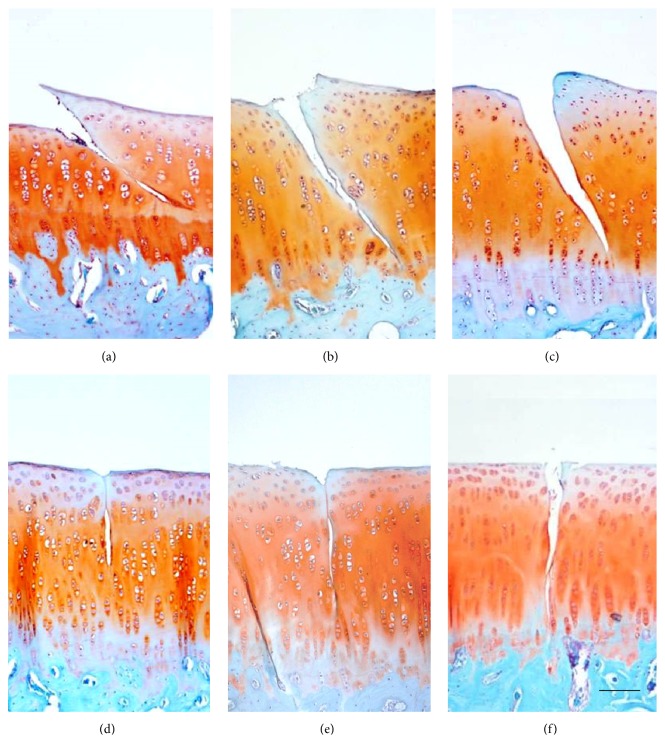
Safranin O staining showed no significant changes from 2 weeks to 20 weeks. (a) 45° crack at 2 weeks. (b) 45° crack at 6 weeks. (c) 45° crack at 20 weeks. (d) 90° crack at 2 weeks. (e) 90° crack at 6 weeks. (f) 90° crack at 20 weeks.

Immunohistochemical results from the animal tests illustrated a 45° crack induced obvious type I collagen expression, with the mean optical density increasing from 0.13 ± 0.07 at 2 weeks to 0.19 ± 0.09 at 20 weeks. However, there was no type I collagen around the 90° crack (Figure 5). Both of type II collagen and proteoglycan expression did not change in 45° or 90° group (Figures 6 and 7).

## 4. Discussion

In the present study, we identified the local tensile stress formation after the cartilage crack through finite element analysis, discovered that the excessive tensile strain could induce change in chondrocyte phenotype, and confirmed the abnormal presence of type I collagen around the crack* in vivo*.

When the joint is damaged, the injury to articular cartilage appears most significant compared with other joint tissues. The damage of cartilage is irreversible and may be the mainly determinant for the development of PTOA [17, 18]. Articular cartilage is continually subjected to repetitive compressive loading during physical activity. Chondrocyte, the only cell type in cartilage, is mechanically sensitive. Appropriate joint loading ensures chondrocytes maintenance of cartilage ECM homeostasis, essential for the healthy functioning of cartilage [19]. We found that the oblique crack with 45° angle could induce the maximum local tensile stress, while the vertical crack induces no tensile stress. This suggests that crack angle could be an important factor for development of PTOA, which could also explain the fact that not all trauma to cartilage resulting in OA [20].

Our study showed that 10% tensile strain significantly decreased the expression level of* COL2A1*,* Acan *and* SOX9*, while induced* COL1A1 *expression. Aggrecan is the predominant proteoglycan in cartilage [21]. Some authors found an upregulation of collagen II mRNA in early degenerative stages corresponding with slightly increased color intensities in immunohistochemical studies for collagen II in deeper layers, which was a symptom of a persisting but initially still intact repair process. Thus, this symptom was followed by a decrease leading to lower type II collagen expression [22]. The downregulation of* COL2A1* and* Acan* may lead to cartilage degeneration and initiation of OA [22]. As the first chondrogenic transcription factor, SOX9 plays crucial roles in chondrocyte differentiation and cartilage formation [23]. Therefore, the downregulation of SOX9 could also influence the development of OA. Type I collagen, which mainly exists in tendon and fibrocartilage, is also a marker of osteoarthritic chondrocytes [24]. 10% tensile strain promoted the mRNA expression of* COL1A1*, which was consistent with animal model analysis.

As tensile stress is one of the causes of cartilage degeneration, it is of value to consider how to minimize the presence of tensile stress in cartilage repair. During the study and application of cartilage tissue engineering, tensile stress should also be paid attention in the design of scaffolds and cartilage materials. Patients receiving rehabilitation should be advised to stay away from any postures or movements that favor local tensile stress formation at the injury sites.

Limitations of this study should also be addressed. We failed to detect the suppression of type II collagen and proteoglycans around cartilage crack* in vivo*. This might be due to the limitation of our animal model. In the animal study, a single scratch was created on the cartilage, and the induced tensile stress was limited around the crack without significant changes of stress formation in the remaining cartilage and subchondral bone. This might lead to a slow pace of cartilage degeneration without obvious change in expression of type II collagen and proteoglycans at early stage. Further studies are needed to verify the findings.

## 5. Conclusions

This study demonstrated occurrence of local tensile stress after cartilage injury, chondrocyte phenotype changes, and formation of type I collagen around the cartilage crack. The findings suggested that the local tensile stress caused by the crack could play important role in cartilage degeneration and initiation of osteoarthritis after joint trauma. This might provide a biomechanical based insight into PTOA pathogenesis and potentially new intervention in prevention and treatment of PTOA.

## Figures and Tables

**Table 1:** Primer sequences of genes used for real-time PCR analysis.

Gene name	Primer sequence (5′-3′)	Primer sequence (3′-5′)
*COL1A1*	GGCAGATGACGCCAACG	CCAGTGTCCATGTCGCAGA
*COL2A1*	AAGCTGGTGAGAAGGGACTG	GGAAACCTCGTTCACCCCTG
*Acan*	CTACACGCTACACCCTCGAC	ACGTCCTCACACCAGGAAAC
*SOX9*	AAGCTCTGGAGACTTCTGAACG	CGTTCTTCACCGACTTCCTCC
*GAPDH*	CTATAAATTGAGCCCGCAGC	ACCAAATCCGTTGACTCCG

## Data Availability

The data used to support the findings of this study are included within the article.
